# Attitudes of dental students towards patients with special healthcare needs: Can they be improved?

**DOI:** 10.1111/eje.12490

**Published:** 2020-01-03

**Authors:** Anita Holzinger, Stefan Lettner, Alexander Franz

**Affiliations:** ^1^ Teaching Center Medical University of Vienna Vienna Austria; ^2^ University Clinic of Dentistry Medical University of Vienna Vienna Austria

**Keywords:** attitudes, dental students, educational programme, evaluation, patients with special health care needs

## Abstract

**Introduction:**

Lack of knowledge and skills as well as negative attitudes towards patients with special healthcare needs may adversely affect the services available to this group. In 2010, a line on the treatment of patients with special healthcare needs has been implemented in the dental curriculum at the Medical University of Vienna, including five seminars and a practical course. In this study, we examine to what extent the programme helps improve attitudes towards persons with special healthcare needs and positively impacts the readiness to engage in treating this clientele.

**Materials and Methods:**

In 2017 and 2018, all students who were in their fourth study year participated in the study. Students' attitudes were assessed before the first seminar, after the last seminar and after the practical course. At all three time points, the same fully structured questionnaire was used, including established instruments for the assessment of emotional reactions and the desire for social distance plus ad hoc questions for assessing students' future intention to treat patients with special healthcare needs. The data were analysed by means of linear fixed models.

**Results:**

At the end of the line devoted to patients with special healthcare needs, students tended less to express negative emotions and showed more positive emotional reactions than before the start of the programme. However, students' social acceptance of such patients and their readiness to engage in treating them did not change significantly.

**Discussion:**

While our programme was able to improve students' emotional reactions to people with special healthcare needs, it proved unable to reduce the desire for social distance and to lower the barrier when it comes to treatment. It is planned to further develop our programme which, hopefully, will then succeed in increasing students’ readiness to treat this clientele.

**Conclusion:**

Improving dental students' emotional reactions to patients with special healthcare needs does not necessarily translate into greater readiness to treat this clientele.

## 1 INTRODUCTION

Access to oral health care for patients with special healthcare needs is a growing challenge. Dental patients with special healthcare needs are those patients whose medical, physical, psychological or social situation sets them apart from other individuals in terms of needs and makes it necessary to modify normal dental routine in order to provide appropriate dental treatment. These patients include people from disadvantaged backgrounds and people lacking social support; further people requiring assistance in daily activities due to physical or developmental disability, mental illness or substance abuse, difficulty seeing or hearing, or certain medical conditions; and finally, people having trouble reading, speaking or understanding the local language.^1^, ^2^ Special Needs Dentistry is defined by the Royal College of Surgeons of Edinburgh as “the specialty in dentistry concerned with the oral health care of patients with special needs for whatever reason including those who are physically or mentally challenged”.^3^

There are numerous studies showing that patients with special needs for dental care are underserved^2^ and, in consequence, show poorer oral health.^4^ This may be due to various reasons located in either the person in need of care or in the provider of dental health care. As concerns the latter, insufficient knowledge of the special healthcare needs of this patient group seems to play a part. Because of a lack of training in behaviour management, communication and treatment planning dentists may not feel prepared and may not feel confident treating these patients. Apart from a lack of clinical proficiency, unfamiliarity with this clientele may foster misconceptions and negative stereotypes, resulting in reluctance to treat patients with special needs for dental care. In fact, several studies have shown that dentists who had undergone training in managing patients with special healthcare needs and who perceived their educational experiences as valuable felt more comfortable with treating this group of patients^5^, ^6^, ^7^ and treated more of these patients compared to those not exposed to this kind of training.^8^ Similarly, students who perceived themselves prepared for the treatment of patients with special needs showed greater future intention to treat this clientele.^9^, ^10^, ^11^

Studies evaluating the effectiveness of education programmes in special needs dentistry, using a pre‐post design, are rather scarce. Sanders et al^12^ found that after completion of an interactive, virtual‐patient module on compact disc, presenting an individual with a developmental disability, students' perceived comfort and knowledge base, has significantly improved. Salama et al^13^ reported that viewing an educational presentation in the form of a DVD was effective in informing dental students and providing them with instructive basic information on patients with special healthcare needs. DeLucia et al^14^ surveyed students immediately before and 1 week, 6 months and 1 year after a lecture on management of patients with intellectual disabilities. They found no significant change over time in current and anticipated comfort in treating this patient group.

The effect of education in dental care of persons with special healthcare needs on students' personal attitudes towards these patients has so far not been studied. In this paper, we will present results of the evaluation of a line on the treatment of patients with special needs which was implemented in the dental curriculum at the Medical University of Vienna in 2010. Up to this moment, no programme of this kind has existed in Austria. We wanted to know to what extent the programme succeeds in reducing negative attitudes, or more specifically, to what extent it helps improve emotional reactions to and increase social acceptance of such people. In addition, we will address the question whether the programme does have a positive impact on students' readiness to engage in treating patients with special needs in their own practice when they will have finished their training in dentistry.

## 2 METHODS

### 2.1 Description of the line on dental care of patients with special needs at the Medical University of Vienna

Participation in the line is mandatory for all dental undergraduate students in the seventh and eighth semester. The programme consists of two components, school‐based education with didactic sessions and a community‐based experience including outreach to institutions serving people with special healthcare needs.

Five seminars are devoted to the dental management of homeless people as well as of patients with paediatric, geriatric, psychiatric and neurological disorders. Each of the seminars lasts 2 hours. They are moderated by two instructors, a dentist and a specialist in the other medical discipline. After a short introduction to the topic by the instructors, the students are divided into small groups of 8‐16 persons, depending on the number of students of the years. Per seminar four patient cases are prepared by the instructors. For each case, handouts with medical history and x‐rays are provided. The way of establishing contact with the patient and the treatment concept are to be worked out both by the dental side and the medical specialty addressed in the respective seminar. At the end of each seminar, spokespersons of the groups present the treatment concept, followed by feedback from the instructors. Through the seminars, students are familiarised with patients whom they will get to know in the subsequent practical course. Seminars serve to prepare students for problems which may arise in the treatment of these patients and which may make necessary adjustment of oral hygiene and conservative prosthetic procedures and which require a more flexible handling of existing therapy concepts. Students learn how to handle communication difficulties which may lead to irritations, tensions and refusal of treatment. Aim is to convey the theoretical background on how to deal appropriately with this particular patient group in order to achieve successful treatment and mutual satisfaction.

The practical course takes place in either a service for children and youth with behavioural abnormalities from socially disadvantaged families, run by the social organisation of the Catholic Church (“Caritas”), or in a public welfare institution for homeless people and people at high risk of poverty, run by the City of Vienna (“Neunerhaus”). While the first provides shared apartments for six to eight children each, supported by professional staff, the latter provides consultation, medical care and shelter. In diagnostic terms, children are suffering from a variety of psychiatric disorders, ranging from attention‐deficit/hyperactivity disorder, learning disorders to autism and related disorders. Among adults, alcoholism and other substance use disorders represented the main health problem. During the practical course, dental interventions are performed by dentists and students are given the opportunity to assist them. Aim of the course is to facilitate contact between students and people with special healthcare needs. Students become more familiar with these people, what may help reducing eventually pre‐existing misconceptions, hopefully resulting in increased understanding and acceptance. Courses are organised in small groups with four students at most. At the beginning of the course, students are introduced to the clientele of the respective services by one of the staff members. At the end, there is a debriefing which serves as basis for a written report each student is supposed to provide.

### 2.2 Study design

In 2017 and 2018, all students in their fourth study year participated in the study (N = 154).

A total of 67 participants were men and 87 women. All students participated at the three assessments, that is there was no attrition of the study group over the observation period.

Students' attitudes were assessed before the first seminar (T1), after the last seminar (T2) and after the practical course (T3). At all three time points, the same questionnaire was used.

The study was approved by the Ethics Committee of the Medical University of Vienna.

### 2.3 Questionnaire

The questionnaire started with the question: “How would you react to people with somatic or mental disabilities or people living in difficult life conditions (homeless, without health insurance coverage, refugee etc), which would make necessary an adaptation of routine dental treatment to their special needs?” We then presented respondents with a scale consisting of 13 items describing possible emotional reactions, asking them to indicate how they would react to such a person. Ten items originated from the Emotional Reactions towards the Mentally Ill (ERMIS) Scale,^15^ representing the emotional dimensions fear, anger and pro‐social reactions. The scale is based on a theoretical concept developed by Dijker et al.^16^ Its three‐dimensional structure has been replicated in several population‐based studies.^17^, ^18^ Three additional items were formulated aimed at representing the aversion such a person may evoke.

Desire for social distance was elicited with a modified version of Link's social distance scale,^19^ including four items that ask respondents how willing they would be to engage in various situations of everyday contact with such a person (moving next door, meeting for a coffee, making friends and inviting to one's home). In numerous studies, the social distance scale has shown good construct and criterion validity.^20^, ^21^

With the help of two ad hoc formulated items, we explored respondents' readiness, after completion of their study in dentistry, to reserve a slot of the working time particularly for the treatment of persons with special needs or whether such patients should better be treated in separate facilities specialised in their care. Throughout the whole questionnaire, answers were given using a four‐point Likert scale with the response categories “do completely agree”,^1^ “do rather agree”^2^, “do rather not agree”^3^ and “do not agree at all”.^4^ In addition, a “don't know” option was offered.

### 2.4 Statistical analysis

Since more than half of subjects answered to at least one question with “don't know” or did not answer at all, exploratory factor analyses were carried out using full information maximum likelihood to deal with missing data. For the remaining analyses, we used multiple imputations by chained equations,^22^ using proportional odds models for imputations of missing items in a Likert scale and logistic regression for binary missing items.

We conducted an exploratory factor analysis with the set of items assessing emotional reactions, determining the number of factors using Horn's parallel analysis.^23^ This procedure yielded three factors, with a minimum eigenvalue of 1.1. We performed varimax rotation of the three factors, resulting in un‐correlated factor scores. Table A1 shows items, rotated factor loadings, eigenvalues and the explained variance of the three factors. Together, they accounted for a cumulative variance of 41%. We termed the first factor “aversion/anger,” the second “uncomfortableness” and the third “pro‐social reactions.” Higher scores indicate stronger emotional reactions. Despite addition of three new items, the factor structure of the instrument remained identical with that of the original scale which has shown good construct, criterion and predictive validity.^15^

As with emotional reactions, we carried out an exploratory factor analysis with the four items exploring respondents' desire for social distance. It yielded one factor with an eigenvalue 2.3 (explained variance 58%). For details, see Table A2. Again, we calculated factor scores, higher scores indicating greater willingness to interact with such people and thus lower desire for social distance. With Cronbach's alpha =.85, the internal consistency of the scale was quite good.

We calculated factor scores resulting from the factor analysis loadings which gave a reasonably close approximation of a normal distribution. Factor scores were used as dependent variable in a linear mixed model, including a random student effect as well as fixed effects of gender and time point.^24^

For single questions, we estimated ordinal cumulative link mixed models using a logit link, that is a proportional odds mixed model. We included time points as fixed effects and a random student effect to account for the structure of the data set.

All computations were performed using R version 3.5.0.^25^

## 3 RESULTS

### 3.1 Attitudes of students towards patients with special healthcare needs at baseline

Emotional reactions of students before and after the seminar as well as after the practical course are reported in Table 1. Already before the start of the curriculum, the majority of students tended to react positively to persons with special needs. For instance, over 95% did not react angrily and were not amused or considered the company of these people embarrassing. Over 80% felt pity for these people and the need to help them and disagreed that these people provoke aversion, fear or annoyance. Relatively, frequently students expressed feelings of uncomfortableness and insecurity and did not feel sympathy for them.

**Table 1:** Emotional reactions to patients with special healthcare needs

	Response category	Before seminar %	After seminar %	After practical course %
I feel uncomfortable	Agree^a^	38.1	22.4	6
	Disagree^b^	62.8	70.5	88
	Don't know^c^	5.1	7.1	5
These persons provoke fear	Agree	10.9	5.1	0
	Disagree	85.3	89.7	96
	Don't know	3.8	5.1	22
I feel insecure	Agree	25.6	19.2	8
	Disagree	69.2	73.7	87
	Don't know	5.1	7.1	3
I find these persons disgusting	Agree	12.8	5.1	9
	Disagree	76.3	87.8	84
	Don't know	10.9	7.1	5
I have an aversion to these persons	Agree	7.7	1.9	6
	Disagree	87.8	94.2	89
	Don't know	4.5	3.9	3
These persons provoke my incomprehension	Agree	5.1	0.6	1
	Disagree	80.8	88.5	93
	Don't know	14.1	10.9	4
I feel annoyed by these persons	Agree	3.2	2.6	1
	Disagree	88.5	92.3	94
	Don't know	8.3	5.1	3
I react angrily	Agree	2.6	0.6	0
	Disagree	95.5	95.6	96
	Don't know	1.9	3.8	2
I feel the need to help	Agree	84.0	85.9	89
	Disagree	9.0	9.0	6
	Don't know	7.0	5.1	4
I feel sympathy for these persons	Agree	48.7	58.3	60
	Disagree	24.4	17.9	17
	Don't know	26.9	23.7	21
I feel compassion for these persons	Agree	84.0	70.5	72
	Disagree	11.5	23.7	23
	Don't know	4.5	5.8	4
I am amused	Agree	02.6	0.0	0
	Disagree	96.8	96.2	96
	Don't know	2.6	3.8	2
The company of these persons is embarrassing	Agree	3.2	1.9	2
	Disagree	94.9	93.0	95
	Don't know	1.9	5.1	4

a Response categories “do completely agree” and “do rather agree” combined.

b Response categories “do rather not agree” and “do not agree at all” combined.

c Response category “don't know” and no answer combined.

However, persons with special healthcare needs were met with considerable reservation (see Table 2). While almost two‐thirds of students would be willing to move next door to these people, only one‐quarter would be ready to invite them to their home. A relatively high percentage of students did not feel able to respond to the questions.

**Table 2:** Social acceptance of patients with special healthcare needs

	Response category	Before seminar %	After seminar %	After practical course %
Make friends with such a person	Agree^a^	44.9	47.4	46.2
	Disagree^b^	21.8	19.2	21.8
	Don't know^c^	33.3	33.3	32.0
Meet such a person for a coffee	Agree	49.4	48.1	48.
	Disagree	24.4	21.8	21.2
	Don't know	26.3	30.1	30.
Invite such a person to your home	Agree	24.4	30.1	25.6
	Disagree	43.6	33.3	42.9
	Don't know	32.1	36.5	31.4
Move next door to such a person	Agree	64.1	60.9	66.0
	Disagree	19.9	17.9	15.4
	Don't know	16.0	21.2	18.6

a Response categories “do completely agree” and “do rather agree” combined.

b Response categories “do rather not agree” and “do not agree at all” combined.

c Response category “don't know” and no answer combined.

At baseline, three‐quarters of students declared themselves willing to reserve a slot of their working time for the treatment of this patient group (see Table 3). On the other hand, almost two‐thirds expressed the view that such patients should better be treated in special facilities.

**Table 3:** Readiness to treating patients with special healthcare needs in one's practice

	Response category	Before seminar %	After seminar %	After practical course %
I'm ready to reserve a slot of my working time for the treatment of such patients	Agree	74.4	80.1	80.1
	Disagree	14.1	9.6	10.9
	Don't know	11.5	10.3	9
Such patients should better be treated in separate facilities specialised in their care	Agree	63.5	57	66.7
	Disagree	19.2	23.1	15.4
	Don't know	17.3	19.9	17.9

Response categories “I completely agree” and “I rather agree” combined.

Response categories “I do rather not agree” and “I do not agree at all” combined.

Response category “I don't know” and no answer combined.

### 3.2 Changes in students' attitudes

As shown in Table 4, across all three dimensions, students' emotional reactions to persons with special needs have improved significantly. At the end of the line, students expressed less uncomfortableness, aversion and anger, and were more likely to show pro‐social reactions. With aversion/anger, significant changes were observed only after the seminar, while with uncomfortableness there was a further improvement after the practical course. The increase in pro‐social reaction was only significant after participating in the practical course.

**Table 4:** Development of emotional reactions to people with special healthcare needs over time

Dimension	Comparison	Estimate	s.e.	Statistic	*P*
Aversion/anger	T1 vs T2	−0.52	0.15	−3.58	<.001
	T2 vs T3	0.02	0.14	0.13	0.899
	T1 vs T3	−0.50	0.14	−3.46	<.001
Uncomfortableness	T1 vs T2	−0.50	0.14	−3.58	<.001
	T2 vs T3	−0.46	0.14	−3.31	0.001
	T1 vs T3	−0.96	0.14	−6.84	<.001
Pro‐social reaction	T1 vs T2	0.04	0.09	0.41	0.681
	T2 vs T3	0.19	0.09	2.08	0.038
	T1 vs T3	0.23	0.09	2.47	0.014

Note

T1 before seminar.

T2 after seminar.

T3 after practical course.

As shown in Table 5, social acceptance has increased over the seminar but decreased at the end of the practical course, resulting in no significant change over the whole time period. Students' readiness to reserve a slot of their working time for the treatment of patients with special healthcare needs did not change significantly. While after the seminar students were less likely as before to share the view that patients with special needs should better be treated in separate facilities, after the practical course agreement with this view has increased significantly, resulting in no significant overall change.

**Table 5:** Development of social acceptance and attitude towards treating patients with special healthcare needs over time

	Comparison	Estimate	s.e.	Statistic	*P* (>|z|)
Social acceptance	T1 vs T2	0.48	0.24	2.05	0.041
	T2 vs T3	−0.45	0.23	−1.94	0.054
	T1 vs T3	0.03	0.23	0.14	0.892
I'm ready to reserve a slot of my working time for the treatment of such patients	T1 vs T2	0.05	0.24	0.21	0.831
	T2 vs T3	−0.15	0.25	−0.62	0.537
	T1 vs T3	−0.10	0.25	−0.41	0.683
Such patients should better be treated in separate facilities specialised in their care	T1 vs T2	−0.54	0.24	−2.27	0.023
	T2 vs T3	0.81	0.25	3.27	0.001
	T1 vs T3	0.27	0.25	1.1	0.271

Note

T1 before seminar.

T2 after seminar.

T3 after practical course.

## 4 DISCUSSION

Summarising our findings, we can state that students at the end of the line devoted to patients with special healthcare needs tended less to express negative emotions and showed more positive emotional reactions than before the start of the programme. Thus, as concerns emotional reactions, our programme showed the expected effect. The most pronounced change was found with uncomfortableness, which was reduced through both the seminar and the practical course. In addition to the exposure to information on how to treat these people, the opportunity to get in personal contact and to become more familiar with them has helped reduce feelings of fear and insecurity. This occurred although the practical course did not meet all requirements proposed by intergroup contact theory^26^ for successful contact. Particularly, the condition of equal status between students and persons with special healthcare needs could hardly be realised. However, as more recent research has shown, positive effects of contact on attitudes can also be achieved in sub‐optimal conditions.^27^ Our results underscore the importance of combining both, school‐based education with didactic sessions and providing students the opportunity to get in personal contact with persons with special healthcare needs. This conclusion seems to be supported by the result of a previous study relying solely on classroom instruction and not including a component providing community‐based experience with special needs patients. In this study, no improvement in students' comfort in treating these patients was achieved.^14^

In contrast to emotional reactions, students' social acceptance of people with special healthcare needs has not changed for the better. At first glance, this result may be disappointing. However, if one has a closer look at the items measuring social acceptance, one observes that all of them refer to private social situations (making friends, meeting for a coffee, inviting to one's own home and moving next door) and not to the professional setting of a dentist. When students expressed fewer negative feelings towards people with special healthcare needs, which hopefully translate into a more adequate treatment of these people, this does not imply that they also should be more willing to engage in private‐social relationships.

Students' readiness to treat these patients in their own practice, once they have finished their training in dentistry, remained unchanged. The same holds true for the view that patients with special needs should better be treated in services specialised in their care. Thus, while the programme seemed to be able to help improve students' reactions to people with special healthcare needs, it proved unable to lower the barrier when it comes to treating them. This contrasts with results of previous studies with dental students as well as with practicing dentists which revealed a positive association between the exposure to education in the treatment of persons with special healthcare needs and the willingness to treat this clientele^5^, ^6^, ^7^, ^8^, ^9^, ^10^, ^11^ (see Introduction). A possible explanation for this discrepancy as well as for the increasing endorsement of specialised services may be sought in students' growing awareness that they were insufficiently prepared for treating this clientele. This perception appears not unjustified as with five seminars and one practical course our programme is rather modest compared to curricula that have already been developed in other countries, particularly in UK, Australia, Canada and the USA^4^, ^28^, ^29^. In view of the growing need for providing dental treatment for patients with special needs,^30^ it is planned to extend the programme. A more comprehensive line including more seminars and more intensive extramural experiences with such patients may have a greater impact on students' attitudes and may also help increase their readiness to treat this clientele. It is highly desirable that similar curricula on special needs dentistry will also be implemented at other medical universities in Austria. This process should be informed by the guideline for curriculum development in Special Care Dentistry developed by the Education Committee of the International Association for Disability and Oral Health (IADH).^31^ Moreover, special needs dentistry should become part of the final state examinations all dental students in this country have to pass in order to get the licence for practicing dentistry. Apart from dentists, also curricula for dental auxiliary students should be developed^32^ as active participation of dental hygiene, dental therapy and oral health therapy practitioners in the care of patients with special needs can also help improve patients' oral health statuses and their access to oral healthcare services.^33^

Our findings must be seen in the light of our study's limitations. First, our study is to be considered as preliminary as we have not used a control group, not to speak of randomization. Second, due to limitations of insurance coverage, students were only allowed to assist to dental interventions but not to perform them themselves. This may have increased student's feeling of being insufficiently prepared for treating patients with special needs. Third, our study focuses exclusively on attitudes which not necessarily may result in corresponding behaviour. Fourth, the last assessment occurred immediately at the end of the programme. We therefore do not know whether the changes observed will persist over a longer time period.

## 5 CONCLUSION

As concerns emotional reactions, the question posed in the title of this paper, namely whether attitudes of dental students towards patients with special needs can be improved, can be answered in the affirmative. However, this does not necessarily mean that students' willingness to treat these patients will also increase.

## CONFLICT OF INTEREST

The corresponding author and the two co‐authors confirm that there is no conflict of interest.

## Appendix 1

**Table A1:** Emotional reactions to patients with special needs: rotated factor loadings

Item	Factor 1 Aversion/anger	Factor 2 Uncomfortableness	Factor 3 Pro‐social reactions
I have an aversion to these persons	**0.660**	0,314	−0.089
I feel annoyed by these persons	**0.624**	0.196	−0.170
These persons provoke my incomprehension	**0.611**	0.049	−0.054
I find these persons disgusting	**0.521**	0.324	−0.066
The company of these persons is embarrassing	**0.489**	0.219	−0.152
I react angrily	**0.475**	0.191	−0.111
I am amused	0.324	−0.041	−0.012
I feel uncomfortable	0.204	**0.794**	−0.078
I feel insecure	0.157	**0.707**	−0.004
These persons provoke fear	0.151	**0.683**	0.090
I feel the need to help	−0.165	−0.131	**0.681**
I feel sympathy for these persons	−0.195	−0.045	**0.532**
I feel pity for these persons	0.051	0.296	**0.519**
Eigenvalue	2.22	2.03	1.11
Cumulative explained variance (%)	0.17	0.33	0.41

Bold values indicate factor loading over 0.4.

**Table A2:** Desire for social distance from patients with special needs. Rotated factor loadings

Please indicate: How willing would you be	Factor 1 “social distance”
To make friends with such a person	**0.838**
To invite such a person to your home	**0.799**
To meet such a person for a coffee	**0.795**
To move next door to such a person	**0.592**
Eigenvalue	2.32
Explained variance (%)	57

Bold values indicate factor loading over 0.4.
